# Conical connection adjustment in prosthetic abutments obtained by different techniques

**DOI:** 10.4317/jced.55592

**Published:** 2019-05-01

**Authors:** Roser Camós-Tena, Tomás Escuin-Henar, Sergi Torné-Duran

**Affiliations:** 1 DDS and MS of Dental and Maxillofacial Rehabilitation and Prosthetics. School of Dentistry of the University of Barcelona (Spain); 2 DMD, PhD. Professor of Oclusion and Prosthodontics, and Director of Dental and Maxillofacial Rehabilitation and Prosthetics. School of Dentistry of the University of Barcelona (Spain); 3 Associate Professor of occlusion and Prosthodontics, and Director of Dental and Maxillofacial Rehabilitation and Prosthetics. School of Dentistry of the University of Barcelona (Spain)

## Abstract

**Background:**

The goal of this study is to compare the misfit (>150µm) generated once the restoration, made by different techniques, is retained to a single conical implant.

**Material and Methods:**

15 internal connection implants (MIS C1 4’20x10mm) are embedded each one perpendicularly to an horizontal surface of the 1x1x2cm poliuretan resin model. The 15 samples obtained are divided in 5 groups depending on the framework process (n=3): 1/casting, 2/overcasting, 3/Ti-base, 4/milling and 5/laser sintering. The cobalt-chromium alloy frameworks are screw-retained to their respective implants to a 30-Ncm torque. Once it is retained the framework to the implant, the next step is to section the sample in half with a diamond saw and verify the correct fit with a stereomicroscope, measuring 4 distances in each side (A, B, C and D). Data is submitted to one-way analysis of variance (ANOVA).

**Results:**

According to equality of variances, significant differences are found in A and B measures (*p*=0,000 in left side in both groups and, *p*=0,007 and *p*=0,001 in right side). In C and D, there are not statistical differences (*p*=0,586 and *p*=0,110 in left side and, *p*=0,101 and *p*=0,089 in right side). However, once it has realized ANOVA test, only C retains the hypothesis and accepts independence.

**Conclusions:**

More samples are needed to conclude reliable statements. However, what it is observed is that milled group presents the best marginal fit. Overcasted and Ti-Base abutments also have good results above casted ones, and, sintered groups has the lowest result. Although, all systems have gaps below 150 µm, so all of them are good options for prosthetic rehabilitation.

** Key words:**Conical implants, abutment connection, misfit.

## Introduction

Despite the different systems of implant-supported restorations, all of them do not always obtain an acceptable levels of fit on the implant-abutment interface (IAI). Achiving a perfect fit is complicated because, during the whole manufacturing procedure, it usually exists a misfit of hundreds of microns (µm) (1).

Passive fit is defined as a condition in which in the absence of external loads, the prosthetic structure does not induce any tension on the implant and its components, and thus not on the surrounding bond (1). Branemark was the first author who gave the first definition of passive fit and he considered that a value of 10µm could not be exceeded. Later, other authors suggested that gaps below 150µm were considered acceptable values of marginal misfit without long-term clinical complications (1-3).

Screw-retained restorations need a special attention in fit terms while cemented restorations are less critic because the luting agent reduces the tension in the implant prosthesis complex (4).

The presence of misfits in implant-retained restorations can generate high levels of stress in the bone-implant interface that can compromise implant osseointegration and generate mechanical and biological complications. In prosthetic terms, it can be observed screw loosening, screw fracture, crestal bone loss and even a loss of osseointegration (3).

Compared with the external connection, internal connection improves the mechanical stability and, when internal connection adopts the form of platform switching, the stress distribution of the IAI is reduced because it contributes to placing the microgap away from the peri-implant bone tissue and to preserv the bone tissue. Moreover, it decreases the screw loosening and screw fracture, the microleakage, the micromovements and the bone resorption (5).

In single implants, it is important to serve the purpose of antirotation because it is more difficult to retain the restoration of single tooth by implants (1).

Our study talks about different metal restorations fabricated by 5 different techniques. First, casted and overcasted frameworks; it is believed that the ones who are overcasted have a better accuracy because of their pre-machined connection (2,4) whereas the casted ones frequently present metal distortions generated by the inconsistency of volumetric expansión and temperature of the material used (including waxes and investments) (4). Secondly, cemented restorations. This type of prostheses needs an intermediate component, the Ti-base, which is cemented onto the metal structure, and an abutment screw that is used for the definitive attachment to the implant. Each Ti-base are designed by a compatible and specific implant system which means that each implant brand has its own Ti-base that guarantees the perfect fit on the IAI (6). Thirdly, the CAD/CAM frameworks (milling and laser sintering). CAD/CAM technology introduces scanning, software design and machining of the prostheses and it demonstrates better fit and quality due to tridimensional digital reproduction than those made by conventional castings (1).

All in all, due to the absolute passivity in a implant-retained restoration is not easy to achieve; the purpose of this *in vitro* study is to compare the most appropriate restoration technique to obtain the lowest value of misfit between the prostheses studied.

From this description, it is hypothesized that it does not exist any difference in fit terms (>150µm) once the abutment (made by different techniques: Casting, overcasting, Ti-Base or CAD / CAM) is screwed to a single conical implant to a 30-Ncm torque.

## Material and Methods

**Figure 1:**
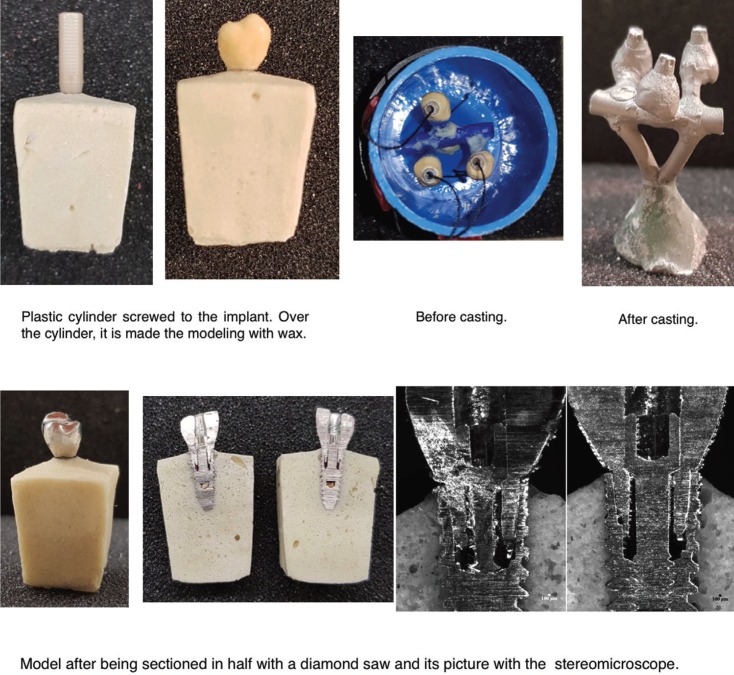
Study procedure example.

Experimental *in vitro* study. 15 implants (C1 Standard Platform, MIS Iberica) were embeded in a polyurethane resin (Modralit 3K) into a silicone (Zetalabor, Zhermack) container. The 15 samples obtained are divided in groups depending on the framework process (n=3). The cobalt-chromium alloy frameworks (Wirobond SG, BEGO) are screwed to a 30 N-cm torque with a torquer. After, the sample is sectioned in half along the vertical axis using a diamond saw (micro grinding machine, EXAKT Technologies). And the gap formation between the different points studied were measured at X100 magnification with a stereomicroscope (Stereo Discovery V12, Zeiss) (Fig. 1).

**Figure 2:**
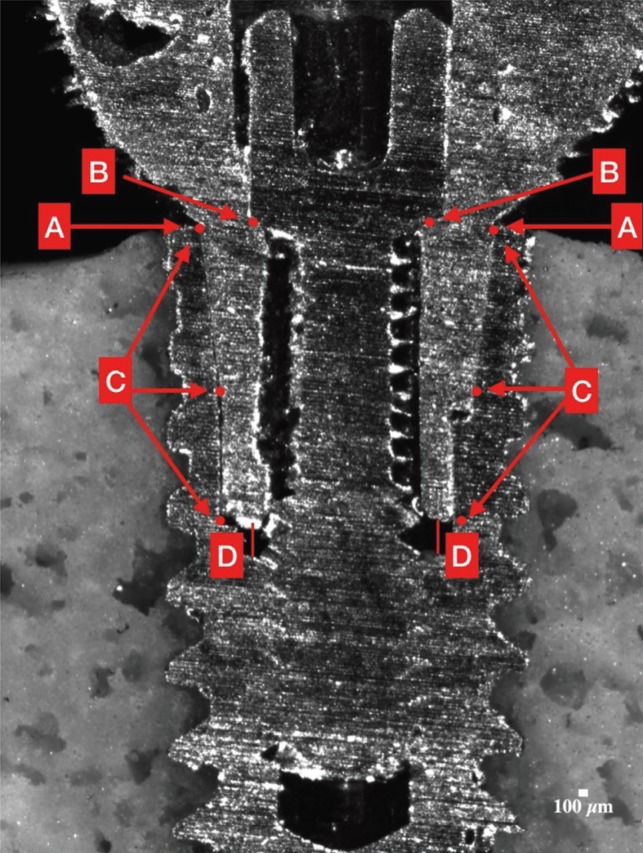
Areas measured marked in red.

To measure the fit, it is used the program ImageJ. It is measured 4 distances in each side, left and right (Fig. 2).

- A: Implant-abutment perimetrical marginal fit (1 point)

- B: Abutment-screw marginal fit (1 point)

- C: Implant-abutment longitudinal marginal fit. A mean of 3 points: basal (=A), middle, apical

- D: Distance between the abutment’s down part and the implant (1 point)

## Results

After the measuring, the obtained results are taken to a database and their statistical analysis is carried out with the package SPSS® Statistics version 22 in the Biostatistics Department of Medicine Faculty at University of Barcelona.

The description of the variable “fit” is made calculating descriptive statistics (e.g. group size, mean, standard deviation) and, the hypothesis about the studied factor has been realized using the one-way analysis of variance (ANOVA). The results are tabulated and represented in tables and error bar graphics. The estimates have been made with a 95% confidence interval for mean and with a significance level (alpha) of 0’05.

From the descriptive results, it is observed that Milled technique has the best fit almost in every point; on the other hand, Peek and Sintered have the lowest. According to A, all techniques have good marginal fit intervals: there is no group, in maximum results, that exceed 150µm.

**Figure 3:**
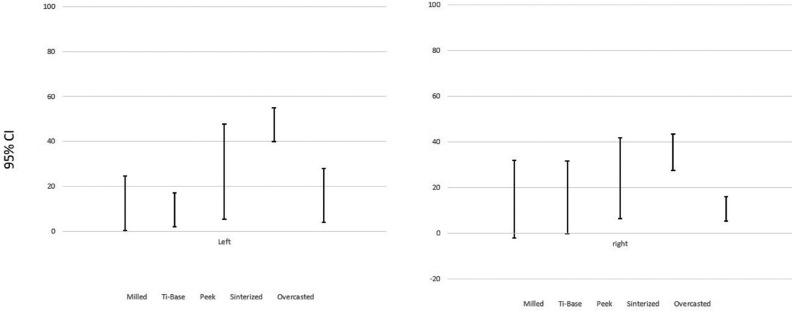
Error bar in C.

**Figure 4:**
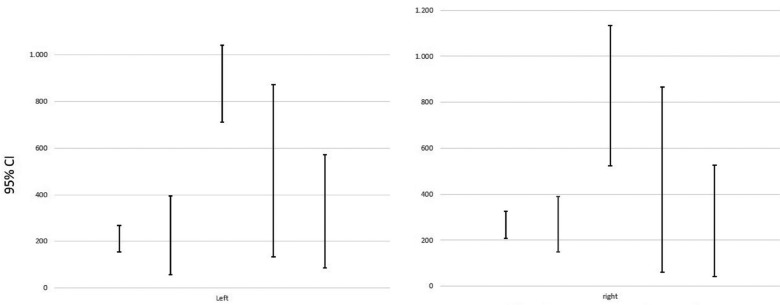
Error bar in D.

About the test of homogeneity of variances ( Table 1), it provides the Levene’s Test to check the assumption that the variances of the five groups are equal. The assumption of homogeneity of variance for these samples differs depending on the zone studied. A and B reject the hypothesis (*p*< 0,05) so it is concluded that these two measures are significantly different; so it exists dependence because there is data dispersion. On the other hand, C and D retain the hypothesis (*p*>0,05) so it is concluded that it does not significantly different between the groups, which it means that the type of restoration chosen does not influence in fit terms. The following error graphics (Figs. 3,4) allows to visually see these results. In both graphics, it is observed that bars are more concentrated between groups so there is more reliability. There is equality of variances between groups and data is less scattered. In C and D there is less dispersion and therefore independence and it is possible to check the assumption of means equality. For this reason, it exists only in C and D the conditions to apply ANOVA test. But just C retains the hypothesis and accepts independence which means that the fit does not depend on the restoration technique.

**Table 1:**
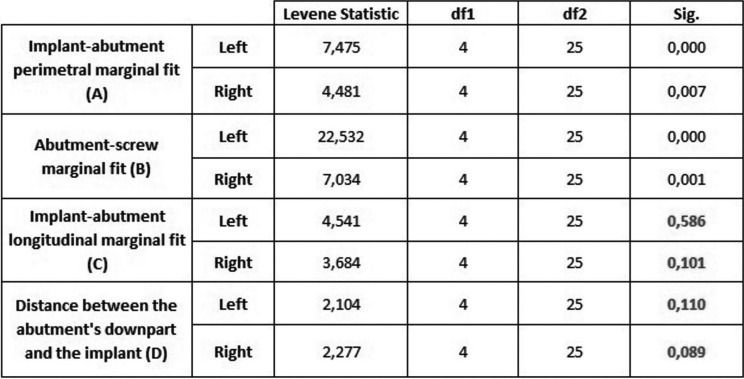
Test of homogeneity of variances.

## Discussion

The choice of implant-abutment connection system is usually based on the professional’s clinical experience.

Different researches explain that internal connections have lower marginal bone loss and it is preferred over external connection implants. However, even being the most accurate implant-abutment connection design (conical internal connection) cannot prevent the endotoxin penetration, but it is still the best kind of connection (6-7). In Karl article, it is compared original versus clone conical abutments, and the study conclusion is that clone abutments look similar to the original components but it has been detected differences and variation in their phsyco-mechanical characteristics by advanced testing methods like 3D inspection. These differences affect reliability and longevity of the restoration, leading to a poor predictability of their outcomes in clinical practice (8).

In the present study, it has been used only conical implants (a cone within a cone). (7) So, the logical reason for using the implant-abutment conical design is to have better prosthetic fit. In aesthetic areas, this connection is also mandatory for the greater stability of peri-implant tissues, especially in thin gingiva biotypes. Acceptable values are below 150µm. (1-2) The results obtained in this study are inside the range of values. To be more exact, all the marginal fit studied do not have more than 60µm.

This kind of connections sealed better because of their friction (7,9). It is important to comment that most common complication is screw loosing (10) so, if there is micro-movement, it interferes the attachment of soft tissue around the implant neck and disrupts the stability of soft tissue that has completed integration. Micro-movement causes micro-gap and therefore a micro-pumping effect which intensifies the leakage of bacteria. Micro-motion inevitably exists at any IAI when there is not fit. However, in Zipprich article, it is said that when it is used a conical abutment there is no micro-motion as long as all parameters of conical self-locking are carried out: cone angle, length of the conical surface, tensions of the tighten screw and manufacturing tolerances. Then, it is ended the microleakage and mechanical damage which produce crestal bone loss around implant neck (11). To reduce the bone resorption, conical and/or platform switching abutments are good connections to the implant; not only reduce the pollution of bacterias but also transfer the harmful microenvironment away from the IAI and close to the implant center (9).

According to the abutment type selected, many articles agreed that casted cylinders have more distortions and less passive fit (2). It is not confirmed for the results obtained in the study the previous statement because the cast results are not ones which have less fit; below them it is found the sintered group. Moreover, polyetheretherketone (PEEK) (12) is a polymer that has many potential uses in dentistry; and, in this study, wax has been replaced by this material. It is necessary to explain that from the many uses that PEEK has, to be casted is not one of these but, for the current study it was the only option found to obtain a full casted abutment.

Turning to the pre-machined abutments, it is observed that they decrease distortions comparing with casted abutments (2). This is because there is a more intimate contact between the pillar platform and the base of the cylinder (2,4). In this study, the overcasted group results are situated in between the casted and milled group.

In CAD-CAM group, it is generally agreed that they allow the best fit. This system eliminates some laboratory steps and human manipulation decrease allowing less errors of fabrication. In this study, the milled group have the best results which confirms what articles revised tell (1,4). But, it is important to add that sintered group (patented by Bego as a Selective Laser Melting), which is CAD-CAM system as well, has the worst results; even worst fit than the casted group. That result should not surprise us because sintered system also has thermal changes during the soldering. New CAD-CAM technic has started to be introduced in prosthetic laboratories. It consists in sintering alloys and then being machined: milling/post-sintering(ML/PS). Some companies like Bioinnovación Dental or Promedent CAD-CAM have started to implement it in Spain. In Bioinnovación Dental they called it Sintex whereas in Promedent, they called it Fullmec. This new system consists on machining the connections, the emergence profile and the chimney access. All the basal abutment is machined and the main advantage is good fit. So, maybe sintered results in the present study are not the best ones but they could be better if after they are milled. This statment should be studied.

Also, Ti-Base group is a good option to choose, it is below milled group and similar to the overcasted group. It is important to add that this abutment is machined but in comparision with the overcasted, it does not have any temperature alteration because it is not put in the oven.

On the consulted bibliography, there is a little controversy between the articles revised and the results of the study. For the articles revised, Peek would have the worst fit results because is the one which is fully casted, causing more metal distortions. But the surprise from this study is that the laser sintering (CAD/CAM group) has even lower fit.

However, it is fair to explain the limitations of the present study. The size of the model(1x1x2) was small to be fixed in the micro-grinding machine and have the half correctly. Another difficulty that has appeared it has been not having a CAD-CAM library specific for the implants used (MIS C1 4’20x10mm); but, without having a library from the commercial company, it is true that CAD-CAM system (milled group) has the best results in terms of fit.

Turning to the 4 distances measures that have been studied, the A (implant-abutment perimetrical marginal fit) is important as it represents the one which could cause screw loosing, micro leakage and micro motion due to the gap and the incorrect fit. If A is within the fit parameters, the restoration has more chances of success. The others are less clinically important in micro leakage terms. However, in tensions terms are quite similar because they are distributed in different points. Although, A is the most important point at a clinical level, but from the statistic results obtained in this study, this point have dependence because it has been rejected the equality of variances so it is not possible to confirm if one restoration method fits better than other.

About the statistic analysis, it should be done with more number of samples for each group to have a more reliable results. But, because of the material budget limits, the group size is very small and the statistic results (in a 95%CI with a 5% error) are not extrapolable. Few samples to conclude any reliable statements. The study can not be considered acceptable, so it is little representative.

## Conclusions

With the limitations of this experimental study, the following can be concluded.

1. Milled group presents statistically the best marginal fit. On the other hand, sintered group, which is a CAD-CAM method too, has the worst results.

2. Overcasted and Ti-Base abutments also have good results above casted ones.

3. All systems have gaps below 150µm (specifically below 60µm), so clinically all of them are good options for rehabilitation.
